# 855. Combined Plasma and High-Dose Vitamin C is a Safe Adjunctive Strategy in Burn Resuscitation

**DOI:** 10.1093/jbcr/irag033.325

**Published:** 2026-04-06

**Authors:** Ana M Reyes, Ian J Brann, Talia R Arcieri, Avery M Hebert, Akshat Sanan, Shevonne S Satahoo, Joyce I Kaufman, Louis R Pizano, Carl I Schulman

**Affiliations:** University of Miami, Miami, FL; University of Miami Miller School of Medicine, Miami, FL; Miami Burn Center, Miami, FL; Miami Burn Center, Miami, FL; Miami Burn Center, Miami, FL; Miami Burn Center, Miami, FL; Miami Burn Center, Miami, FL; Miami Burn Center, Miami, FL; Miami Burn Center, Miami, FL

## Abstract

**Introduction:**

Fresh frozen plasma (FFP) and high-dose vitamin C (HDVC) and are both used as adjuncts to crystalloid-based burn resuscitation and have been associated with reduced fluid requirements and edema. However, no study has evaluated their combined use in burn patients. We hypothesized that combination therapy with FFP and HDVC would reduce fluid requirements without increasing complications compared with either strategy alone.

**Methods:**

Adults with ≥40% TBSA admitted to a single burn center from 2015-2025 were retrospectively reviewed. Patients who received FFP, HDVC, or both within 24 hours were included; those who died within 48 hours were excluded. Fluid requirements (mL/kg/%TBSA), urine output (mL/kg), and rates of acute kidney injury (AKI), dialysis, ARDS, compartment syndrome, and ICU mortality were compared between groups using Kruskal-Wallis or chi-square tests.

**Results:**

Of 73 patients (67% male, mean age 43 ± 15 years, mean TBSA 64 ± 16%), 19 (26%) received FFP and HDVC, 17 (23%) FFP alone, and 37 (51%) HDVC alone. The FFP + HDVC group had the largest burns (70 ± 16% TBSA) compared to the FFP-only (66 ± 15%) and HDVC-only (59 ± 16%) groups (*p*=.048). Crystalloid and total fluid volumes were similar across groups, while urine output in 48 hours was greatest in the HDVC-only group (Table 1). Rates of AKI, dialysis, ARDS, compartment syndrome, and ICU mortality did not differ significantly between groups.

**Conclusions:**

Combination therapy with FFP and HDVC was associated with fluid requirements and complication rates comparable to either adjunct alone, despite patients in the combination group having significantly larger burns. These findings suggest that it is safe to administer FFP and HDVC in combination, though future studies with cohorts more closely matched for burn severity are required to clarify potential risks and benefits.

**Applicability of Research to Practice:**

The use of combined FFP and HDVC during burn resuscitation is likely safe, with outcomes comparable to FFP or HDVC alone.

**Funding for the study:**

N/A.

